# A QTL genome scan of the metabolic syndrome and its component traits

**DOI:** 10.1186/1471-2156-4-S1-S96

**Published:** 2003-12-31

**Authors:** Matthew B McQueen, Lars Bertram, Eric B Rimm, Deborah Blacker, Susan L Santangelo

**Affiliations:** 1 Department of Epidemiology, Harvard School of Public Health, Boston, Massachusetts, USA; 2 Genetics and Aging Unit, Department of Neurology, Massachusetts General Hospital, Harvard Medical School, Charlestown, Massachusetts, USA; 3 Department of Nutrition, Harvard School of Public Health, Boston, Massachusetts, USA; 4 Gerontology Research Unit, Department of Psychiatry, Massachusetts General Hospital, Harvard Medical School, Charlestown, Massachusetts, USA; 5 Psychiatric and Neurodevelopmental Genetics Unit, Department of Psychiatry, Massachusetts General Hospital, Harvard Medical School, Charlestown, Massachusetts, USA; 6 Center for Human Genetics Research, Massachusetts General Hospital, Harvard Medical School, Charlestown, Massachusetts, USA

## Abstract

**Background:**

Because high blood pressure, altered lipid levels, obesity, and diabetes so frequently occur together, they are sometimes collectively referred to as the metabolic syndrome. While there have been many studies of each metabolic syndrome trait separately, few studies have attempted to analyze them *combined*, i.e., as one composite variable, in quantitative trait linkage or association analysis. We used genotype and phenotype data from the Framingham Heart Study to perform a full-genome scan for quantitative trait loci underlying the metabolic syndrome.

**Results:**

Heritability estimates for all of the covariate-adjusted and age- and gender-standardized individual traits, and the composite metabolic syndrome trait, were all fairly high (0.39–0.62), and the composite trait was among the highest at 0.61. The composite trait yielded no regions with suggestive linkage by Lander and Kruglyak's criteria, although there were several noteworthy regions for individual traits, some of which were also observed for the composite variable.

**Conclusion:**

Despite its high heritability, the composite metabolic syndrome trait variable did not increase the power to detect or localize linkage peaks in this sample. However, this strategy and related methods of combining correlated individual traits deserve further investigation, particularly in settings with complex causal pathways.

## Background

Coronary artery disease (CAD) and related cardiovascular diseases are genetically complex and heterogeneous. Several identified risk factors for CAD, including diabetes mellitus, arterial hypertension, hypercholesterolemia, and obesity also show strong and at least partially independent genetic components [1]. To date, efforts to identify the genes underlying these traits have had only limited success, and only a few positive findings have been replicated (e.g., [2-4]).

A particular complication to the identification of novel CAD genes is the high correlation among cardiovascular risk factors. In fact, because high blood pressure, altered lipid levels, obesity, and diabetes so frequently occur together, they are sometimes collectively referred to as the metabolic syndrome or syndrome X (MSX [5]). Because MSX was initially defined for clinicians, the syndrome and its various component traits were dichotomized, although each is more informatively viewed as a quantitative trait. While there have been many studies of each MSX trait separately [2,6], only a few studies have considered the syndrome itself, either as a whole or broken into factors, in quantitative trait linkage or association analysis (e.g., [7-9]).

Our study utilizes genotype and phenotype data from the Framingham Heart Study, made available for the Genetic Analysis Workshop 13 (GAW13), to search for quantitative trait loci (QTL) underlying MSX in this large, carefully followed sample. Specifically, we defined five MSX-related quantitative traits across the life-span (systolic blood pressure (SBP), triglycerides (TGL), high-density lipoprotein cholesterol (HDL), blood sugar (BS), and body mass index (BMI), calculated their heritabilities, and performed whole-genome scans on each. We then combined the individual quantitative traits into a composite quantitative trait, the metabolic syndrome score (MSS), and calculated its heritability and scanned the genome for linkage.

## Methods

### Description of the data set

From the data provided for Problem 1 in the GAW13 data set (Framingham Heart Study), we selected the 1617 individuals between the ages of 30 and 69 with at least one examination cycle in which there was complete data for all five component measures (SBP, TGL, HDL, BS, and BMI). These age limits were chosen because our preliminary analyses, consistent with the cardiovascular literature, suggested that heritability was greatest in this interval, and because the data tended to be sparse at lower and higher ages. We used all available complete observations (examination cycles with complete data on all 5 traits) from age 30 to 69 for each individual, and developed a life-span measure for each trait, as described below. The number of *complete *observations available for each subject varied from one (*n *= 116) to five (*n *= 524), with a mean of 3.7 observations per subject. Of these 1617 individuals, 1313 also had genotype data available.

### Estimation of the phenotypes

For each trait, we began by transforming as needed to achieve an approximately normal distribution [10]. SBP, HDL, and BMI (calculated as [weight (kg)/height^2 ^(m)] were approximately normally distributed, so we used the raw values. TGL was log-transformed to better approximate a normal distribution. BS was markedly skewed and leptokurtotic, so we followed the procedure used by Meigs et al. [11] (ranking the measurement across all observations, and then standardizing the resulting ranks by subtracting out their mean and dividing by their standard deviation). Note that all available blood sugar measurements, whether fasting or not, were included in order to maximize the number of individuals with complete data in each family. We also ran all analyses using only those measurements explicitly noted to be fasting (all measurements for Cohort 2, and measurements 10–12 [for which we took the average in order to minimize missing data] for Cohort 1). This resulted in only minor differences from the results reported here, perhaps related to our use of ranks rather than actual observed values. In addition, it should be noted that the *fasting *BS values reported for exams 10–12 for Cohort 1 were actually higher on average than those reported for the other *non-fasting *exams.

For each of these variables, we then adjusted for alcohol and smoking in linear regression analyses of each trait performed separately for males and females at each age. Age was considered in 1-year bands to increase the precision of our analyses, and to ensure that each subject contributed only once to these values. Alcohol consumption was categorized as 0, 0.1–12, 13–25, 26–40, and >40 g/day to allow for non-linear effects, while the number of cigarettes smoked per day was entered as a continuous trait.

The residuals from these linear regression analyses were then standardized, again within groups defined by gender and age, by subtracting the age and gender specific mean and dividing by the age and gender-specific standard error. Since the resultant residuals were approximately normally distributed, the standardization process yielded trait values that followed a N ~ (0, 1) distribution. We then averaged each gender- and age-standardized phenotype over all available visits to obtain a "life-span" estimate for each subject. We also calculated a corresponding average age at which subjects contributed data.

To generate an MSS for each of the subjects' available observations, we combined the standardized residuals for each component trait *prior to the calculation of life-span averages *as follows: [MSS = SBP + TGL + BS + BMI - HDL]. HDL was subtracted from the score because it is protective against CAD, and tends to be *inversely *correlated with the other component traits; thus, *lower *HDL values correspond to a higher MSS. These resulting sums were then averaged to create a lifespan MSS. The MSS as calculated yields a lifetime score for each individual that is an indication of how his or her MSS deviates from the age and gender mean.

### Estimation of heritability and linkage analysis

Heritability estimates were calculated, controlling for average age and gender at which subjects contributed data, for each of the component traits, and for the MSS composite trait, using the variance-components methods implemented in SOLAR [12]. We then performed two-point and multi-point quantitative trait linkage analysis, also using SOLAR. Individuals without genotype information were included in the linkage analyses, because the proportion of marker alleles shared identically by descent was estimated for both genotyped and non-genotyped individuals in pedigrees.

## Results

### Descriptive statistics

Descriptive statistics for raw values of each of the component traits, prior to covariate adjustment and standardization procedures, are provided in Table 1. These figures demonstrate a broad range consistent with published data on the Framingham Study. MSS, as a sum of standard deviations from the mean for each of the component traits, is not readily interpretable beside these raw values, so it is not included in Table 1: it exhibited a mean of 0.15, a standard deviation of 2.7, and a range of -7.3 to 11.8.

### Heritability estimates

Heritability estimates for the individual component traits were all fairly high, ranging from 0.39 to 0.62, and MSS was at the high end of this range at 0.61 (Table 2). All heritability estimates were highly statistically significant (*p *< 0.00001).

### Multi-point linkage analysis

**Figure 1:**
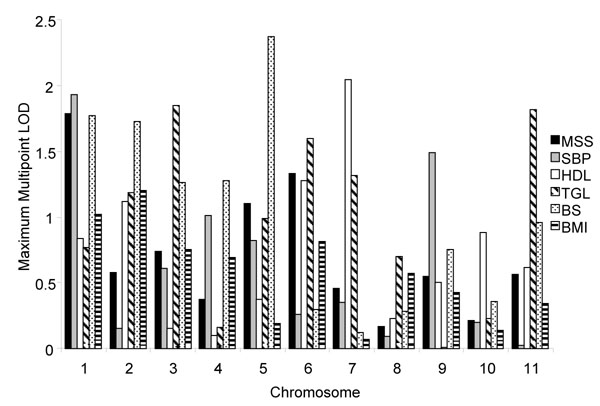
Maximum multi-point LOD scores on chromosomes 1–11 for each of the phenotypic traits.

**Figure 2:**
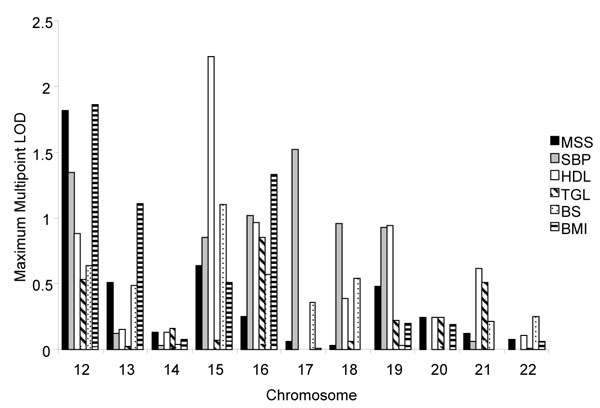
Maximum multi-point LOD scores on chromosomes 12–22 for each of the phenotypic traits

For each phenotype, the maximum multi-point LOD score and its cytogenetic and chromosomal location are presented in Table 2. While we observed no statistically significant linkage for any trait, there were two locations with suggestive linkage by Lander and Kruglyak's criteria [13]: a LOD of 2.4 for BS on chromosome 5pter, and a LOD of 2.3 for HDL on chromosome 15p13-q11. There was also a LOD of 2.1 for HDL on chromosome 7p14. There was essentially no linkage signal for MSS in any of these locations, but MSS did give LOD scores of 1.82 on 12q13 (a signal also seen with BMI) and 1.79 on chromosome 1q42 (a signal also seen with SBP). The overall results are summarized in Figures 1 and 2. The two-point results (data not shown) were consistent with the multi-point findings.

## Discussion

In our full-genome scan for novel QTLs underlying MSX and its component traits in the Framingham Heart Study, we found evidence for at least two 'suggestive' loci-a LOD of 2.3 for HDL on 15p13-q11 and a LOD of 2.4 for BS on 5pter, and one locus just short of this threshold-a LOD of 2.1 for HDL on 7p14. Interestingly, these three peaks do not map close to linkage signals observed in recent studies of MSX, BS, or HDL in the Framingham data (e.g. [11,14,15]) or in a variety of independent samples (e.g. [1,6,16]). However, the location of the maximum signal we found for MSS-a LOD of 1.82 on 12q13-mapped relatively close to locations found in other studies involving type 2 diabetes-related phenotypes (e.g. [17-19]). In addition, we did observe more minor signals that were consistent with prior reports, particularly for BS (e.g., the LOD of 1.77 on chromosome 1q42 for BS coincides with earlier results from the Framingham sample [11]). Discrepancies between our findings and prior studies may be due to differences in defining the individual quantitative phenotypes (e.g., transformation, averaging of longitudinal data), different methods of generating combined phenotypes (e.g., crude sum vs. factor analysis vs. other weighting schemes), different inclusion criteria (e.g., using a broad but not complete age range and including only examination cycles with complete data on all five traits), or chance variation. Of note, while averaging across visits within subjects was intended to incorporate some of the richness of the longitudinal data, it may actually have led to a decrease in some signals.

## Conclusions

Despite its high heritability, the relatively crude estimate of a composite metabolic syndrome variable (MSS) did not increase our ability to detect or localize linkage peaks in this sample. Not surprisingly, the only two noteworthy signals for MSS appear to be driven by linkages to its component traits (BMI and SBP on chromosome 12q13, and BS on chromosome 1q42). Nonetheless, we feel that similar strategies, bucking the current trend toward disaggregation of complex phenotypes, may merit further exploration in this and other settings with complex causal pathways marked by extensive correlation among intermediate causes. If this approach is to succeed, it will be critical to develop weighting schemes that more accurately represent the contribution of each trait to the overall syndrome.

## Figures and Tables

**Table 1:** Descriptive statistics of each of the component phenotypes.

**Trait**	**Mean**	**Std. Deviation**	**Minimum**	**Maximum**
SBP (mm Hg)	122	8	86	195
HDL (mg/dl)	50	13	17	93
TGL (mg/dl)	120	84	15	1205
BS (mg/dl)	97	18	61	302
BMI (kg/m^2^)	26	4.6	16	49

**Table 2:** Heritability estimates, maximum multi-point LOD scores, and their location for each quantitative phenotype.

**Trait**	**Heritability**	**Max LOD**	**Chr.**	**Location (cM)**	**Marker**^A^
MSS	0.61	1.82	12q13	63	GATA5A09
SBP	0.48	1.93	1p31	97	GATA61A06
HDL	0.62	2.27	15p13-q11	1	GATA81F03
TGL	0.56	1.85	3p26	9	GATA22G12
BS	0.39	2.37	5pter	0	028xb12
BMI	0.51	1.86	12q21	85	GATA26D02

^A^Closest marker to the observed multi-point LOD score peak.
